# Ictal laughter induced by repetitive transcranial magnetic stimulation in stroke patients: a case series

**DOI:** 10.3389/fpsyt.2026.1925476

**Published:** 2026-09-04

**Authors:** Shusheng Jiao, Xiuhong Xu

**Affiliations:** Department of Neurology, Bethune International Peace Hospital, Shijiazhuang, Hebei, China

**Keywords:** adverse effect, case series, gelastic seizure, ictal laughter, repetitive transcranial magnetic stimulation (rTMS), stroke rehabilitation

## Abstract

**Background:**

Repetitive transcranial magnetic stimulation (rTMS) is a well-established neuromodulation technique for post-stroke motor rehabilitation with a favorable safety profile. However, its safety in patients with acute/subacute cerebral infarction remains incompletely characterized, particularly regarding unusual behavioral responses. Gelastic seizures, characterized by stereotyped, inappropriate laughter without a corresponding emotional trigger, are a rare form of focal epilepsy typically associated with hypothalamic hamartomas or other structural brain lesions. Their induction by rTMS has been exceptionally reported.

**Case presentations:**

We report two cases of acute ischemic stroke in right-handed middle-aged males who developed stimulus-locked ictal laughter during low-frequency (1 Hz) rTMS applied to the left primary motor cortex (M1) for upper limb rehabilitation. Case 1 was a 48-year-old male with right temporo-parieto-occipital infarction who developed recurrent, uncontrollable ictal laughter during the third rTMS session. The laughter was site-specific and reproducible, elicited only when the coil was positioned over the left M1 and ceasing upon repositioning to the prefrontal area. Post-stimulation video-electroencephalography (VEEG) with bipolar montage analysis revealed sporadic single sharp waves over the right anterior temporal (F8), bilateral dorsolateral prefrontal cortex (F3, 4) during wakefulness. Seven months later, upon re-challenge, the patient promptly reported an urge to laugh, leading to immediate cessation. Case 2 was a 42-year-old male with a right lacunar infarct in the globus pallidus and internal capsule who developed ictal-like laughter during the fifth rTMS session, preceded by a premonitory urge to laugh during the fourth session. Consciousness was fully preserved in both patients, and each reported post-ictal dysphoria. Discontinuation of rTMS resulted in complete symptom resolution, with no recurrence at follow-up.

**Conclusions:**

To our knowledge, this is the first report of rTMS-induced ictal laughter in stroke patients. These observations expand the spectrum of rTMS-associated adverse effects, challenge the assumption that low-frequency protocols are uniformly safe in the post-stroke population, and underscore the need for clinical vigilance in patients with structural brain lesions.

## 1 Introduction

Acute ischemic stroke is a leading cause of disability worldwide, characterized by the sudden onset of focal neurological deficits due to cerebral artery occlusion. Clinical manifestations depend on the affected vascular territory and may include hemiparesis, aphasia, sensory loss, and visual field defects. Current treatment strategies focus on rapid revascularization (intravenous thrombolysis and endovascular thrombectomy) in the hyperacute phase, followed by comprehensive rehabilitation to promote functional recovery (1). Repetitive transcranial magnetic stimulation (rTMS) is a non-invasive neuromodulation technique that has gained widespread acceptance in post-stroke neurorehabilitation (2). By delivering repetitive magnetic pulses to specific cortical regions, rTMS modulates neuronal excitability and induces neuroplastic changes, thereby facilitating functional recovery of motor, language, and cognitive functions (3). The safety profile of rTMS has been well established through extensive clinical studies and consensus guidelines (4). Common adverse events in stroke patients include transient headache, scalp discomfort, and dizziness. Headache is the most frequently reported side effect, with an incidence of approximately 36% in active rTMS groups compared to 30% in sham groups (5). Less common but transient symptoms such as local pain, paresthesia, and muscle twitching have also been described (6). The most serious adverse event associated with rTMS is the induction of seizures, although the overall incidence is low, estimated at <0.1% for low-frequency protocols (4). In stroke populations, contemporary large-scale studies have reported no cases of epilepsy or exacerbation of neurological symptoms in subacute ischemic stroke patients receiving high-frequency rTMS (7). However, the safety profile may differ in patients with structural brain lesions, such as acute or subacute stroke, where cortical hyperexcitability and disrupted inhibitory networks may lower the seizure threshold (8).

Gelastic seizures, characterized by stereotyped, inappropriate bouts of laughter without a corresponding emotional trigger, are a rare form of focal epilepsy (9). They are most commonly associated with hypothalamic hamartomas (10, 11), but can also arise from extrahypothalamic foci including the frontal lobe (12), temporal lobe (13), parietal lobe (14), occipital lobe (15), and cingulate gyrus (16). The term “ictal laughter” is often used interchangeably to describe laughter occurring during a seizure, whether or not it constitutes the sole or predominant seizure manifestation. While gelastic seizures are well recognized in the context of structural brain pathology, their induction by rTMS has been exceptionally documented. To our knowledge, only one previous case report has described rTMS-induced excessive laughter, which occurred in a child with autism spectrum disorder receiving high-dose intermittent theta-burst stimulation (17).

The primary motor cortex (M1) is a frequent target for rTMS in stroke rehabilitation, where low-frequency stimulation (≤1 Hz) is often applied to the contralesional hemisphere to reduce interhemispheric inhibition and facilitate motor recovery (18). Although low-frequency rTMS is considered safe and even potentially anticonvulsant (4), the effects of stimulating M1 in the context of acute/subacute cerebral infarction remain incompletely characterized, particularly with respect to unusual behavioral or emotional responses. The possibility that M1 stimulation could inadvertently engage the neural networks subserving laughter and emotional expression has not been previously explored.

Herein, we report two cases of acute ischemic stroke in right-handed middle-aged males who developed reproducible, stimulus-locked ictal laughter during low-frequency rTMS applied to the left M1 for upper limb motor rehabilitation. These cases highlight a previously underrecognized adverse effect of rTMS, underscore the importance of clinical vigilance during stimulation in stroke patients, and provide novel insights into the neural circuitry of laughter. We discuss the potential mechanisms, clinical implications, and safety considerations, and propose directions for future research.

## 2 Case presentations

### 2.1 Case 1

A right-handed 48-year-old male, with a three-year history of poorly controlled type 2 diabetes mellitus, a 30-pack-year smoking history, occasional alcohol consumption, and a family history of hypertension and diabetes in his father, presented to our emergency department on July 21, 2024, with a one-day history of acute-onset left homonymous hemianopia, blurred vision, and progressive weakness and numbness in his left upper limb. Onset of symptoms was upon waking. His body mass index (BMI) was 26.8 kg/m².

#### 2.1.1 Neurological examination

On admission, his blood pressure was 138/85 mmHg, pulse 96 bpm. He was alert, oriented, and fluent. Cranial nerve examination revealed a left homonymous hemianopia with intact pupillary light reflexes. Motor examination demonstrated left upper extremity weakness (proximal 4/5, distal 3+/5) with normal tone. Sensation and coordination were intact. The National Institutes of Health Stroke Scale (NIHSS) score was 3 (visual field 2, motor arm 1).

#### 2.1.2 Laboratory and imaging findings

**Figure 1:**
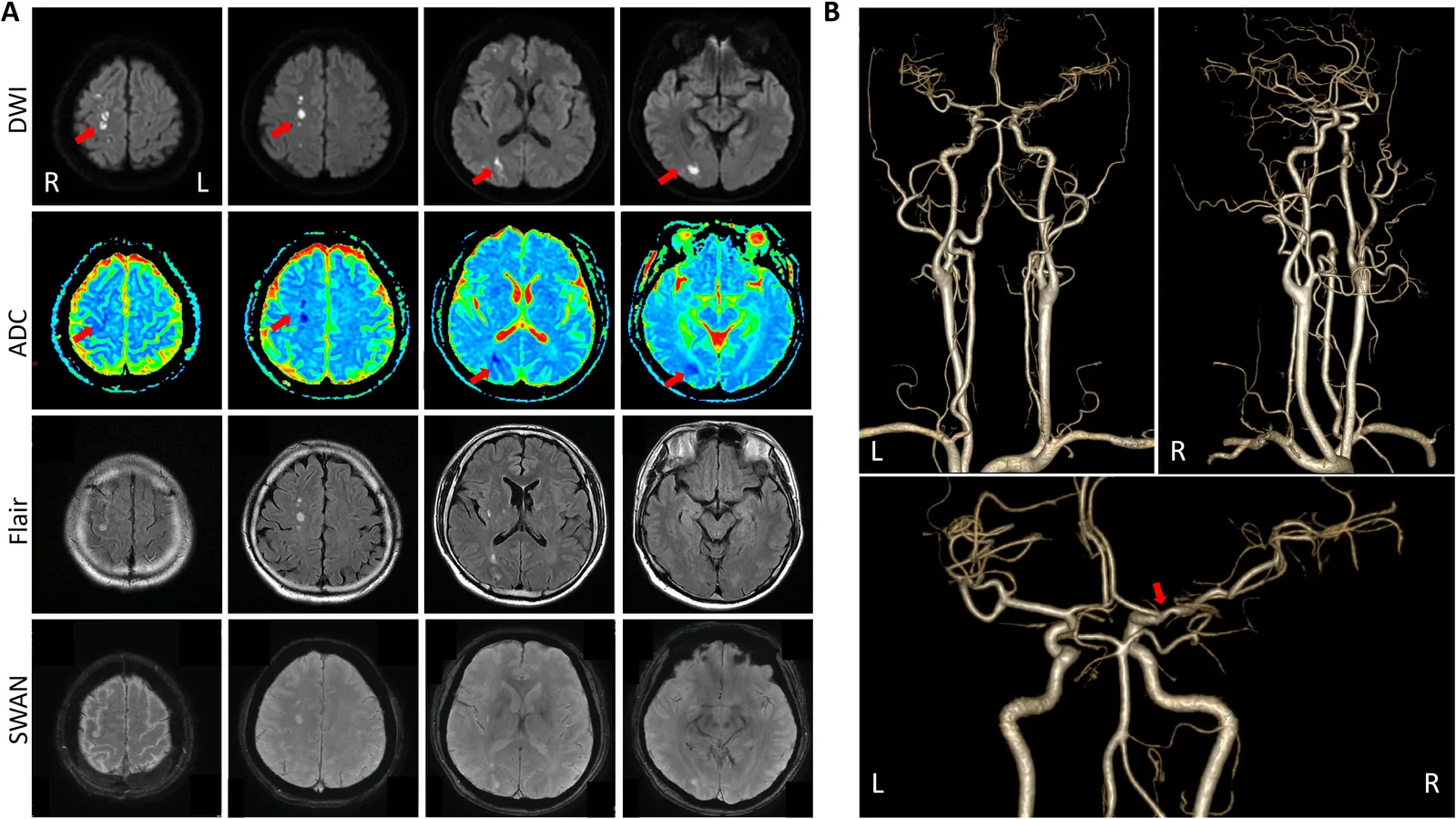
Neuroimaging findings from the first admission (Case 1). **(A)** Axial brain MRI sequences demonstrating acute ischemic lesions. The DWI (upper left) and the corresponding ADC (upper right) map reveal multiple patchy hyperintense signals with reduced ADC values in the right temporal, parietal, and occipital cortices and subcortical white matter, consistent with acute infarction (red arrows). The FLAIR (lower left) confirms corresponding parenchymal hyperintensities, while SWAN (lower right) shows no evidence of microbleeds or paramagnetic deposition. **(B)** The CTA of the craniocervical vessels. The upper panels display a posterior view (left) and a left anterior oblique view (right), illustrating the overall configuration of the anterior and posterior circulations, as well as the origin and course of the vertebral arteries and bilateral internal carotid arteries. The lower panel, a magnified view of the right MCA, demonstrates mild luminal narrowing at the proximal M1 segment (red arrow), indicative of early atherosclerotic change. No evidence of large-vessel occlusion or high-grade stenosis is observed in the major intracranial arteries. MRI, magnetic resonance imaging; DWI, diffusion-weighted imaging; ADC, apparent diffusion coefficient; FLAIR, fluid-attenuated inversion recovery; SWAN, susceptibility-weighted angiography; CTA, computed tomography angiography; MCA, middle cerebral artery.

Initial laboratory tests were notable for hyperglycemia (10.68 mmol/L), hyperlipidemia (low-density lipoprotein cholesterol, LDL-C 3.67 mmol/L), and hyperhomocysteinemia (27.3 μmol/L). Emergent non-contrast head computed tomography (CT) showed no hemorrhage. Brain MRI-DWI (diffusion-weighted imaging) revealed acute multifocal infarcts in the right temporal, parietal, and occipital cortices and subcortical white matter (**Figure 1A**). Magnetic resonance angiography (MRA) and CT angiography (CTA) demonstrated mild stenosis of the right middle cerebral artery (MCA) M1 segment and a hypoplastic right vertebral artery (**Figure 1B**). Cardioembolic workup, including Electrocardiograph (ECG), transthoracic echocardiography, and transcranial Doppler (TCD) bubble study, was unremarkable. An extensive autoimmune and prothrombotic panel was also negative. The final diagnosis was acute ischemic stroke (TOAST classification: large-artery atherosclerosis).

#### 2.1.3 Treatment and rTMS course

The patient was treated with dual antiplatelet therapy (aspirin 100 mg + clopidogrel 75 mg daily), high-intensity statin (atorvastatin 40 mg), and antidiabetic agents. On the third day of hospitalization, a low-frequency (1 Hz) rTMS protocol was initiated for motor rehabilitation over the left M1 (the ipsilesional M1 for the affected limb), using a figure-of-eight coil with a 70% resting motor threshold (RMT) for 20 minutes daily, based on evidence from systematic reviews supporting the efficacy and feasibility of rTMS in the acute phase of stroke (19). The rTMS was delivered using a YRD CCY-I device (Yiruide, Wuhan, China). The coil was positioned over the left M1 (hand area) as the motor rehabilitation target. The stimulation parameters were: low-frequency (1 Hz) mode, stimulation intensity 70% of resting motor threshold (RMT, which was 87.5% of maximum stimulator output), stimulation duration 12 seconds, 12 pulses per train, inter-train interval 2 seconds, 86 repetitions, total stimulation time 20 minutes, total pulses 1,032. The resting motor threshold was determined as the minimum stimulator output intensity required to elicit a motor evoked potential of ≥50 μV in the contralateral first dorsal interosseous muscle in at least 5 of 10 consecutive trials.

**Figure 2:**
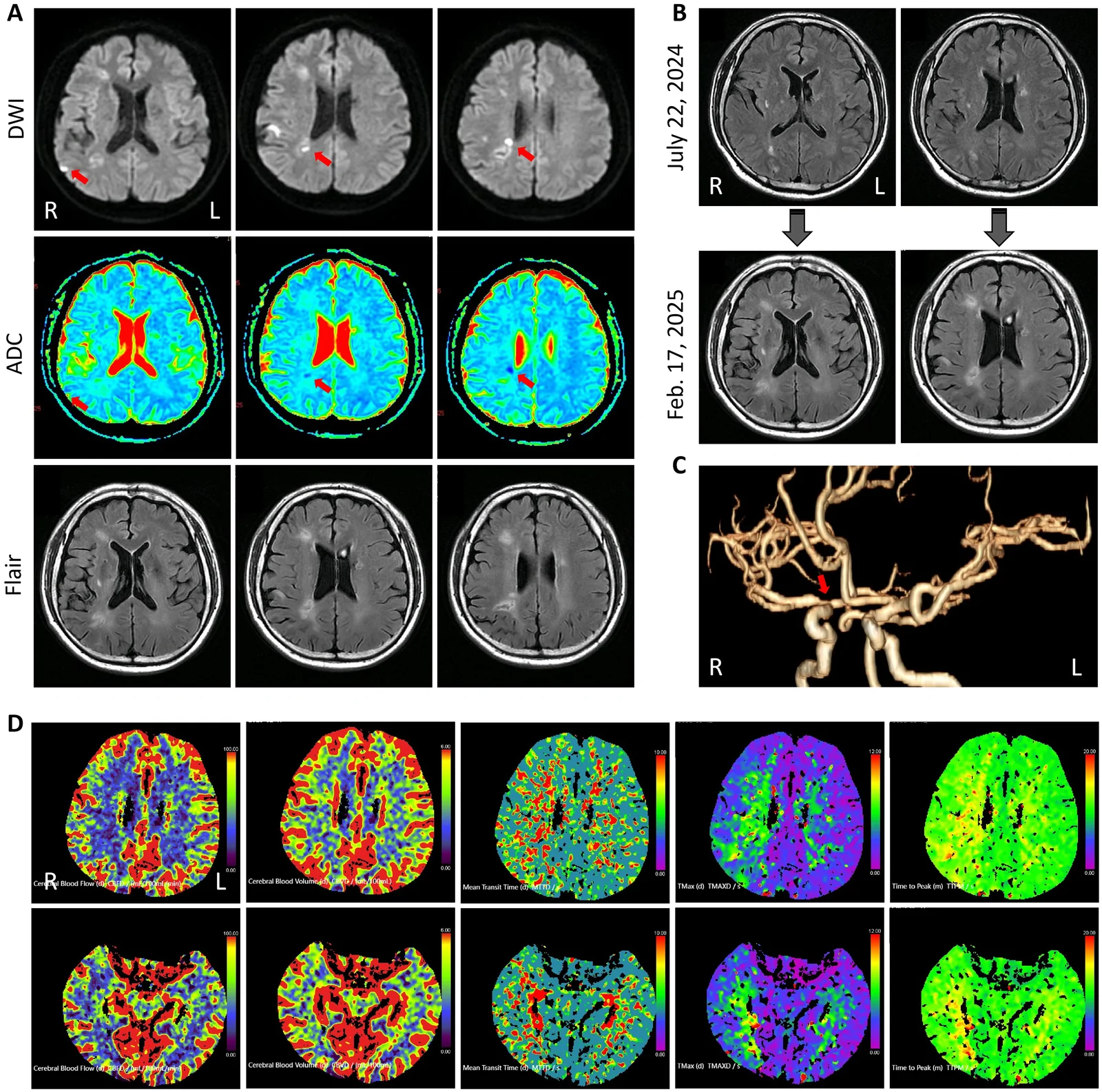
Neuroimaging findings from the second admission (Case 1). The patient was readmitted seven months after the initial stroke with recurrent left-sided weakness. **(A)** Brain MRI reveals multiple patchy acute infarcts involving the deep white matter of the right frontal and occipital lobes (red arrows). The panels are arranged from top to bottom as DWI, ADC, and FLAIR, respectively. **(B)** Comparison of FLAIR sequences between the two admissions demonstrates a marked interval increase in white matter hyperintensities, indicating progression of chronic microvascular pathology. **(C)** The CTA shows severe luminal stenosis of the right MCA at the M1 segment (red arrow), representing significant atherosclerotic progression compared to the prior study. **(D)** The CTP maps reveal prolonged MTT and TTP in the cortex and subcortical white matter of the right frontal, parietal, occipital, and temporal lobes, corresponding to the MCA territory, while CBV and CBF remain relatively symmetric to the contralateral side. These findings are consistent with hemodynamic compromise and perfusion insufficiency without overt irreversible ischemic core expansion. FLAIR, fluid-attenuated inversion recovery; CTA, computed tomography angiography; MCA, middle cerebral artery; CTP, CT perfusion; MTT, mean transit time and TTP, time-to-peak; CBV, cerebral blood volume; CBF, cerebral blood flow.

During the third rTMS session, the patient developed sudden, uncontrollable episodes of inappropriate laughter. He remained fully conscious, able to follow commands, and reported a complete lack of a corresponding emotional state. The patient described the laughter as “completely involuntary” and “not funny at all,” reporting that he felt “embarrassed and confused” during the episodes. The laughter episodes lasted between 5 and 40 seconds (**Figure 2A**), followed by a brief period of dysphoria, restlessness, and a transient sensation of increased limb weakness. When the coil was repositioned to the left prefrontal cortex, the laughter ceased. Upon reapplication to the left M1, the laughter promptly recurred, demonstrating a clear stimulus-site-specific relationship (see **Supplementary Video 1**, Supplementary). While the patient was actively laughing, a real-time video-electroencephalography (VEEG) was attempted but was confounded by significant artifact from the rTMS device. Immediately after the rTMS session, a 1-hour video-EEG was performed. It revealed sporadic single sharp waves over the right anterior temporal region (F8), the right frontal region (F4, right dorsolateral prefrontal cortex), and the left frontal region (F3, left dorsolateral prefrontal cortex) during wakefulness, as identified through bipolar montage analysis (**Figure 2B**). After joint interpretation by senior neurologists and an experienced EEG technologist, these findings were considered suggestive of post-ictal cortical irritability but were non-diagnostic of definitive epileptiform discharges, as they lacked the consistent morphology, field distribution, and temporal evolution characteristic of true epileptic activity. rTMS was discontinued after the third session (on the day the laughter episodes occurred). No further rTMS was administered during the remainder of his hospitalization, and no further laughter episodes were observed during the admission or at follow-up.

**Figure 3:**
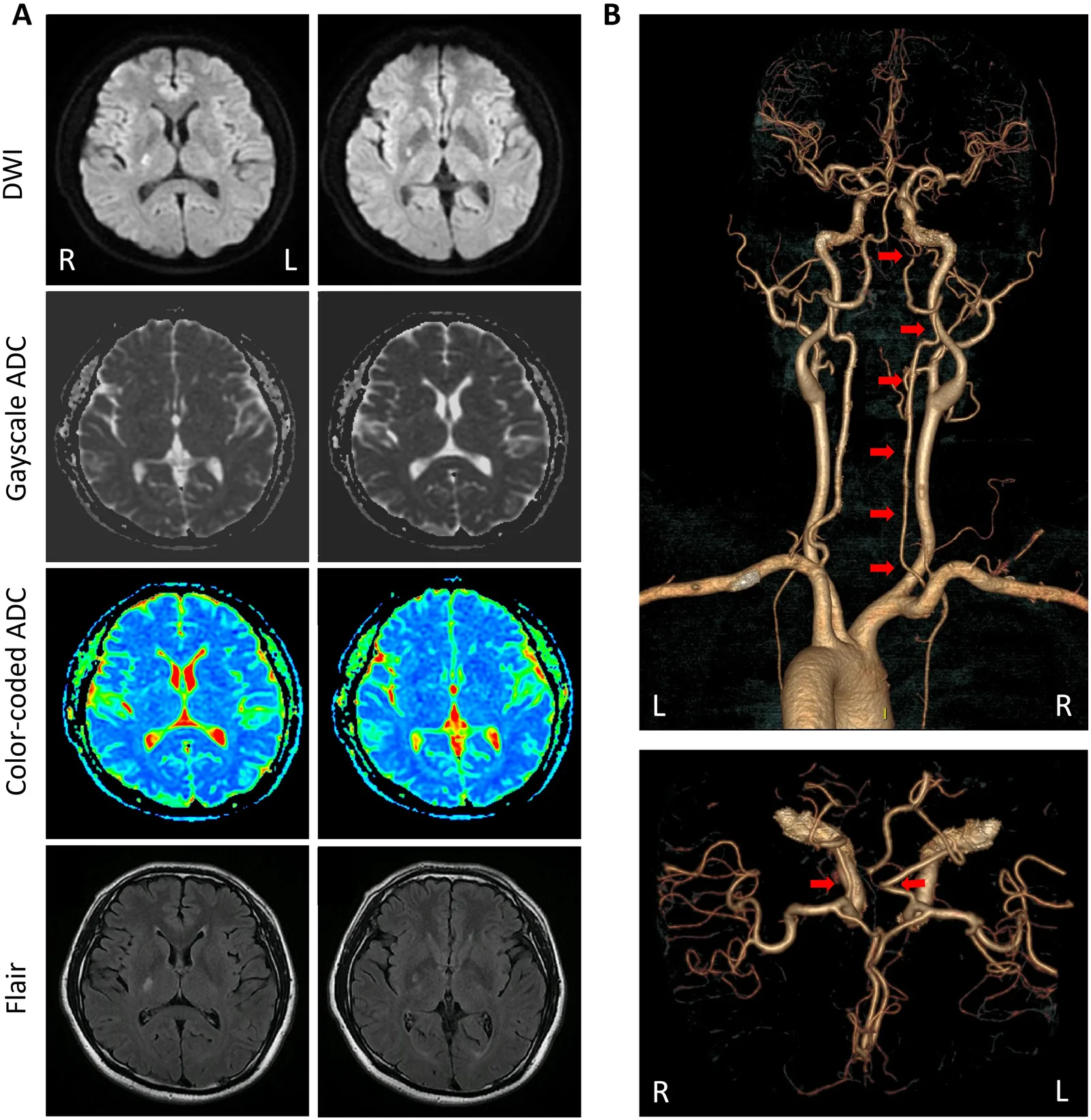
Neuroimaging findings of Case 2. **(A)** Axial brain MRI sequences demonstrating an acute lacunar infarct in the right globus pallidus and the posterior limb of the internal capsule. The panels are arranged from top to bottom as DWI, grayscale ADC map, color-coded ADC map, and FLAIR, respectively. **(B)** The CTA of the craniocervical vessels. The upper panel reveals diffuse atherosclerotic changes throughout the anterior and posterior circulations, with notable hypoplasia of the right vertebral artery and complete occlusion at the V4 segment (red arrowhead). The lower panel demonstrates bilateral fetal-type PCAs, indicating a variant posterior circulation anatomy with dominant supply from the internal carotid arteries. DWI, diffusion-weighted imaging; ADC, apparent diffusion coefficient; FLAIR, fluid-attenuated inversion recovery; CTA, computed tomography angiography; PCAs, posterior cerebral arteries.

Second Admission (February 15, 2025): Seven months later, he was readmitted with a recurrent left-sided weakness. Brain MRI showed new acute infarcts in the right frontal and occipital lobes (**Figure 3A**), with a significant progression of white matter hyperintensities on FLAIR (**Figure 3B**). CTA revealed progression of the right MCA stenosis to severe (**Figure 3C**), and CT perfusion (CTP) showed a prolonged mean transit time (MTT) and time-to-peak (TTP) in the right MCA territory, consistent with hemodynamic compromise (**Figure 3D**).

On the 6th day of hospitalization, in a controlled setting, rTMS was again applied to the left M1 using an identical protocol to the previous admission. Within 5 minutes of stimulation, the patient reported a strong urge to laugh. Stimulation was immediately terminated, and no laughter episode occurred. No further rTMS was administered during the remainder of his hospitalization. He declined interventional treatment and was managed conservatively. One month after discharge, the patient underwent middle cerebral artery stenting. At the 3-month follow-up, he had made a near-complete recovery. No further gelastic episodes have been reported at follow-up. The patient is still being followed up.

### 2.2 Case 2

A right-handed 42-year-old male (BMI 24.5 kg/m²), with no significant past medical history, no smoking or alcohol use, and no family history of cerebrovascular or cardiovascular disease, was admitted on July 19, 2024, with a 3-day history of progressive left-sided weakness and a 1-day history of new-onset speech difficulty following sleep deprivation.

#### 2.2.1 Neurological examination

Blood pressure was 139/92 mmHg. He was alert but presented with motor aphasia. Motor examination revealed 4/5 strength in the left upper and lower limbs, with decreased pinprick sensation on the left. NIHSS score was 3 (aphasia 1, motor arm 1, sensory 1).

#### 2.2.2 Laboratory and imaging findings

**Figure 4:**
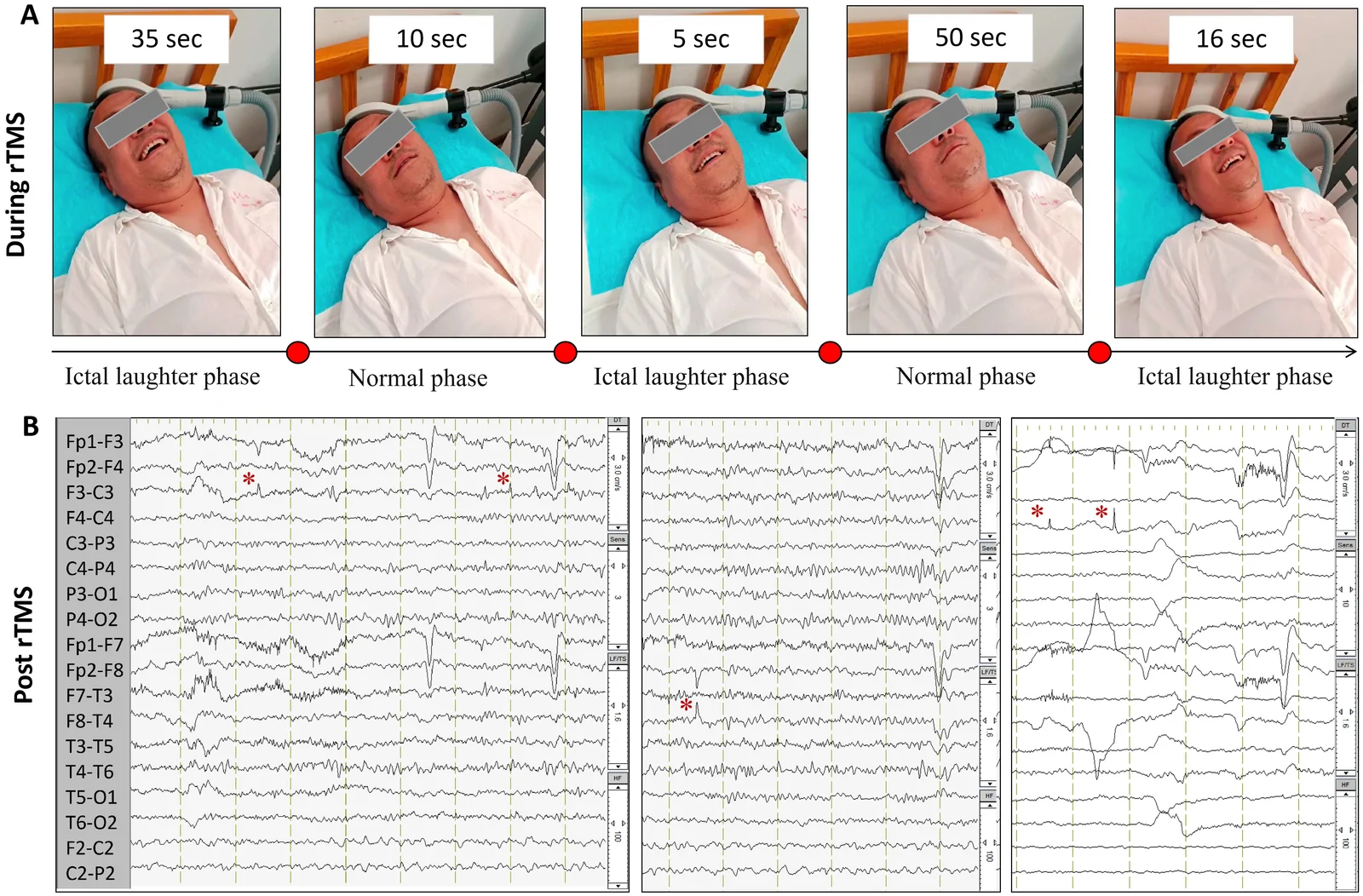
Video still captures during rTMS-induced ictal laughter episodes and post-rTMS video-electroencephalography (VEEG) findings (Case 1). **(A)** Sequential video still frames obtained during the third rTMS session (labeled “during rTMS”), demonstrating the temporal evolution of ictal laughter episodes from baseline to laughter onset and peak expression. **(B)** Post-stimulation VEEG recording (bipolar montage) obtained immediately after the rTMS session (labeled “post rTMS”). Red stars indicate sporadic single sharp waves observed over the right anterior temporal region (F8), the right frontal region (F4, right dorsolateral prefrontal cortex), and the left frontal region (F3, left dorsolateral prefrontal cortex). The dynamic clinical semiology is provided in **Supplementary Video 1**.

Laboratory investigations were notable for hypertriglyceridemia (2.77 mmol/L), hyperuricemia (582 μmol/L), and hypokalemia (3.40 mmol/L). Brain MRI-DWI showed an acute right-sided lacunar infarct in the globus pallidus and posterior limb of the internal capsule (**Figure 4A**). CTA revealed diffuse atherosclerotic changes, hypoplasia of the right vertebral artery with occlusion of the V4 segment, and bilateral fetal-type posterior cerebral arteries (**Figure 4B**). Workup for cardioembolic and autoimmune etiologies was negative. The diagnosis was acute ischemic stroke (TOAST: small-vessel occlusion).

#### 2.2.3 Treatment and rTMS course

The patient was managed with standard antiplatelet and lipid-lowering therapy. One week after symptom onset, an identical low-frequency rTMS protocol over the left M1 (1 Hz, 70% RMT) to that used in Case 1 was initiated for motor rehabilitation. During the fourth session, the patient reported a transient urge to laugh during stimulation, with a lowered laughter threshold for several hours post-session, but he did not report it to the medical team. During the fifth session, he developed clear, uncontrollable gelastic episodes, lasting 5–30 seconds. Consciousness was preserved throughout, and he had full recall of the events, describing them as involuntary and devoid of any humorous trigger. The patient reported that the laughter “came out of nowhere” and that he had “no control over it.” He described feeling “restless and uneasy” after the episodes and noted that “even though it looked like laughing, it didn’t feel like laughing at all”. He reported post-ictal dysphoria and restlessness. To further confirm the stimulus-site-specific relationship found in Case 1, the coil was similarly repositioned from the left M1 to the left prefrontal cortex and back, yielding an identical pattern of laughter cessation and recurrence. The patient declined video recording, formal video-EEG, and additional investigations, including functional MRI (fMRI). The rTMS therapy was ceased on the same day, and no further laughing episodes were reported. He was discharged on July 28, 2024, with no recurrence at follow-up. At the 3-month follow-up, the patient had achieved a near-complete functional recovery, returned to normal daily activities and employment, and scored 0 on both the modified Rankin Scale (mRS) and the NIHSS. The patient remains under follow-up.

## 3 Discussion

We report two cases of ictal laughter induced by low-frequency rTMS applied to the primary motor cortex (M1) in patients with acute ischemic stroke. Several features of these cases merit attention. First, the gelastic episodes were stimulus-site-specific and reproducible: in two cases, laughter was elicited only when the coil was positioned over the left M1, and ceased immediately upon repositioning to the prefrontal area, with prompt recurrence upon reapplication to the original site. Second, the phenomenon occurred during low-frequency (1 Hz) rTMS—a protocol generally considered to have a lower seizure risk compared with high-frequency stimulation (20)—and in the context of acute/subacute cerebral infarction, a condition that may lower the seizure threshold (8). Third, to our knowledge, this is the first report of rTMS-induced ictal laughter or suspected gelastic seizures in stroke patients. While rTMS-related adverse events are well-documented, including headache, scalp discomfort, and rarely seizures (21, 22), gelastic phenomena have only been described in a single case of a child with autism spectrum disorder receiving high-dose intermittent theta-burst stimulation (17). Thus, our cases expand the spectrum of rTMS-associated adverse effects and highlight a previously underrecognized phenomenon in the stroke rehabilitation population. Clinically, these observations underscore the need for vigilance during rTMS therapy in patients with structural brain lesions. Scientifically, they provide a unique window into the neural networks subserving laughter and emotional expression, offering an opportunity to probe cortico-subcortical connectivity in the human brain.

Several lines of evidence from the EEG findings in Case 1 lend partial support to an ictal or peri-ictal origin of the suspected laughter episodes. First, the anatomical distribution of the sharp waves—involving the right anterior temporal (F8), bilateral dorsolateral prefrontal cortex (F3, 4)—is noteworthy, as these areas have been consistently implicated in previously reported cases of gelastic seizures arising from extrahypothalamic foci (23). The temporal lobe, particularly the right hemisphere, is the most frequently reported seizure onset zone in gelastic epilepsy without hypothalamic hamartomas, while frontal lobe involvement, including the anterior cingulate and supplementary motor areas, has also been well documented (24). Second, the temporal association between the laughter episodes and the emergence of sharp waves in the immediate post-ictal period provides a chronological link that supports a causative relationship between the stimulation-induced cortical activation and the subsequent electrographic abnormalities. Third, the presence of multifocal sharp wave activity across both hemispheres suggests engagement of a distributed cortico-subcortical network rather than a single isolated focus, which is consistent with the current understanding of gelastic seizures as network-level phenomena involving fronto-limbic-cingulate circuits (11). Fourth, the finding that sharp waves were observed during wakefulness—rather than exclusively during drowsiness or sleep, where benign variant waveforms are more common—increases their clinical relevance. Taken together, while these sharp waves do not meet definitive criteria for epileptiform discharges, their anatomical concordance with known gelastic seizure foci, their temporal proximity to the ictal events, and their multifocal distribution collectively strengthen the inference that the laughter episodes were ictal or peri-ictal in nature, rather than purely behavioral or affective phenomena.

The topographic distribution of the sharp waves observed in Case 1—involving the right anterior temporal (F8), bilateral dorsolateral prefrontal cortex (F3, 4) —provides important clues regarding the neural networks engaged by M1 stimulation. The frontal and temporal localization aligns with the two major neural systems subserving laughter: an emotional network anchored in limbic and paralimbic structures (including the amygdala, hypothalamus, and anterior cingulate) and a volitional network involving the frontal operculum and motor cortices (25, 26). Tractography studies have demonstrated that the primary motor cortex (M1) projects to brainstem motor nuclei through the internal capsule and is interconnected with the presupplementary motor area (pre-SMA), which in turn connects to both the pregenual anterior cingulate cortex (pACC) and the frontal operculum—key nodes of the emotional and volitional laughter networks, respectively (25). Thus, stimulation of M1 could potentially engage the broader laughter network through trans-synaptic spread along these established pathways. The presence of sharp waves over the right temporal and frontal regions—areas previously implicated in gelastic seizures arising from extrahypothalamic foci (27)—is consistent with the hypothesis that M1 stimulation propagated excitation to these distant but functionally connected nodes. The right frontocentral region, in particular, has been identified as a seizure onset zone in cases of gelastic seizures triggered by motor activity (27), suggesting a shared network between motor execution and laughter generation. Furthermore, the multifocal distribution of sharp waves across both hemispheres, rather than a single isolated focus, supports the concept of gelastic seizures as network-level phenomena involving distributed cortico-subcortical circuits, as has been proposed in the context of hypothalamic hamartoma-related epileptogenesis.

The precise mechanism by which rTMS over M1 precipitates ictal laughter remains speculative, but several interconnected pathways may be implicated. Ictal laughter, classically associated with hypothalamic hamartomas, can also arise from extrahypothalamic foci, including the frontal lobe, insula, cingulate gyrus, and temporal lobe (24, 28). The primary motor cortex is not traditionally considered a core node of the “laughter network”; however, it has reciprocal connections with premotor, prefrontal, and cingulate regions, which in turn project to limbic and paralimbic structures. We hypothesize that low-frequency rTMS applied to the left M1, in the setting of an ipsilesional or contralesional stroke, may induce aberrant spread of cortical excitation along these anatomical pathways, effectively “kindling” a network that subserves gelastic behavior. Notably, in both patients, the ictal laughter emerged only after multiple rTMS sessions (the third and fifth sessions, respectively), suggesting that a cumulative effect or progressive lowering of the threshold for aberrant cortical excitation may be required to precipitate this phenomenon. This temporal pattern raises the possibility that repeated stimulation gradually facilitates network-level hyperexcitability, potentially through activity-dependent synaptic plasticity or progressive disinhibition within cortico-limbic circuits. This is consistent with the concept that focal cortical stimulation can engage distant but functionally connected networks (11). Supporting this, functional connectivity studies in gelastic epilepsy have implicated a cortico-subcortico-cerebellar network, with involvement of the somatosensory cortex (11). The fact that our patients had right-hemisphere lesions—which may disrupt interhemispheric inhibition and facilitate pathological synchrony—further supports a network-based mechanism. Both patients were right-handed, with stimulation applied over the language-dominant left hemisphere; whether this hemispheric dominance contributed to the gelastic phenomena remains to be elucidated. Additionally, the acute phase of stroke is associated with peri-infarct hyperexcitability, altered GABAergic tone, and disrupted transcallosal balance (29), which may lower the threshold for rTMS-induced after-discharges.

We considered whether the gelastic episodes might represent an affective switching phenomenon, as rTMS has been reported to modulate mood in stroke patients, particularly improving post-stroke depression and apathy (30–32). However, several features argue against a purely emotional origin in our patients. First, both patients explicitly denied any corresponding humorous or emotional trigger and described the laughter as “involuntary” and “not feeling like laughing at all.” Second, the stimulus-site-specific and reproducible nature of the episodes—demonstrated by coil repositioning—is more consistent with focal cortical stimulation engaging a specific neural network than with a generalized affective response. Third, the post-ictal dysphoria and the presence of post-stimulation sharp waves in Case 1 support an ictal or peri-ictal phenomenon rather than a primary emotional dysregulation. While rTMS can influence mood through prefrontal stimulation, particularly in the context of post-stroke depression, the features of our cases align more closely with network-level hyperexcitability than with affective switching.

These findings carry several clinically significant implications. First, they challenge the assumption that low-frequency rTMS is uniformly safe in stroke patients. Although the overall incidence of rTMS-induced seizures is estimated at <0.1% for low-frequency protocols (20), our cases suggest that patients with acute/subacute ischemic lesions may represent a higher-risk subgroup. This aligns with earlier safety studies indicating that stimulation parameters safe for healthy volunteers may pose increased risks in chronic stroke populations (21, 22). Second, the phenomenon was site-specific and reproducible, allowing for rapid identification and cessation. Clinicians should be aware that unusual emotional or behavioral responses—including inappropriate laughter, a lowered laughter threshold, or an urge to laugh—may herald an impending ictal event. In Case 2, the patient reported a premonitory urge to laugh during the fourth session, which was not acted upon; this symptom could serve as an early warning sign. Third, the absence of frank convulsive activity does not preclude an ictal origin; ictal laughter is a recognized form of focal epilepsy (14), and their recognition requires clinical vigilance rather than reliance solely on EEG findings, which may be non-diagnostic. Fourth, our cases underscore the importance of real-time patient monitoring and active questioning about subjective experiences during rTMS sessions, particularly in the first week of treatment when cortical excitability may be dynamic. Fifth, from a practical standpoint, we recommend that if gelastic or pre-ictal symptoms emerge, stimulation should be immediately discontinued, and the treatment plan should be reassessed, potentially with modification of stimulation parameters (e.g., lower intensity, shorter train duration, or alternative target site). In Case 1, laughter recurred upon re-challenge seven months later, suggesting that this susceptibility may persist beyond the acute phase. Finally, these observations highlight the need for updated safety guidelines that specifically address rTMS use in stroke patients, particularly those with cortical involvement, and for clearer recommendations on monitoring and managing ictal-like phenomena.

Several limitations should be acknowledged. First, this is a case series of only two patients, which limits the generalizability of our findings. Furthermore, the lack of video documentation and video-EEG recordings in Case 2 limited our ability to fully characterize the semiology and electrographic features of the ictal laughter in this patient. Similarly, the absence of an interictal EEG recording in Case 1 precluded comparison with the post-stimulation findings, limiting our ability to characterize baseline EEG abnormalities. Second, we were unable to obtain definitive ictal EEG recordings during the ictal laughter due to technical interference from the rTMS device, and the post-stimulation VEEG in Case 1 showed only sporadic single sharp waves. Thus, while the clinical features are highly suggestive of gelastic seizures, we cannot conclusively prove their epileptic nature. Third, we did not perform advanced neuroimaging, such as resting-state fMRI or diffusion tensor imaging, which would have allowed us to map the structural and functional connectivity from the stimulation site to potential laughter-generating networks and thereby strengthen the mechanistic interpretation of our findings. Furthermore, the use of manual localization—rather than neuro-navigation or fMRI-guided targeting—may have introduced variability in stimulation site accuracy. Fourth, the stimulation parameters used (1 Hz, 70% resting motor threshold, 20 minutes) were within standard safety guidelines (20), and we did not systematically test other protocols or sites to determine whether the effect is frequency-, intensity-, or site-dependent. Fifth, the lack of a control group or sham stimulation means we cannot exclude a placebo or conditioned response, although the reproducibility and site-specificity argue against this. Sixth, patient-reported outcomes and long-term follow-up are limited to telephone interviews; formal neuropsychological or psychiatric assessments were not conducted to evaluate for persistent affective changes. Finally, the possibility of unmasking a pre-existing, subclinical epileptic tendency cannot be entirely excluded, although neither patient had a history of seizures or epilepsy.

Future research should aim to clarify the neural mechanisms underlying rTMS-induced gelastic phenomena. Future studies employing image-guided targeting techniques, such as neuro-navigation or fMRI-guided rTMS, are warranted to precisely delineate the relationship between stimulation site and gelastic responses. In parallel, functional neuroimaging modalities—including resting-state fMRI and simultaneous EEG-fMRI—could further elucidate the neural networks engaged during and after M1 stimulation, and identify whether specific connectivity patterns predispose individuals to this adverse effect. Additionally, systematic dose-finding and site-mapping studies in stroke patients are needed to establish safer stimulation parameters and to define the therapeutic window for rTMS in this population. Prospective studies with larger sample sizes and standardized EEG monitoring would help establish the true incidence of this phenomenon. From a translational perspective, if rTMS can reliably and safely provoke laughter in a controlled manner, it might offer a unique tool for studying the neurobiology of humor, emotion, and social behavior, and potentially even for therapeutic neuromodulation of mood disorders. However, such applications remain speculative and would require extensive safety and efficacy validation. Finally, we encourage the reporting of similar cases to build a more comprehensive understanding of this rare but clinically important adverse event, which may ultimately inform the revision of rTMS safety guidelines for stroke rehabilitation.

## 4 Conclusion

We report, to our knowledge, the first case series of rTMS-induced ictal laughter in patients with acute ischemic stroke, demonstrating that low-frequency (1 Hz) rTMS over the primary motor cortex may provoke reproducible, stimulus-site-specific gelastic episodes in the context of stroke-related cortical hyperexcitability. These observations expand the spectrum of rTMS-associated adverse effects and challenge the assumption that low-frequency protocols are uniformly safe in the post-stroke population. Clinicians should recognize that unusual emotional responses—including inappropriate laughter, a lowered laughter threshold, or a premonitory urge to laugh—may herald an impending ictal event, warranting immediate stimulation cessation and reassessment of the treatment plan. Further research is warranted to elucidate the underlying neural mechanisms and to establish safer stimulation parameters for stroke rehabilitation.

## Data Availability

The original contributions presented in the study are included in the article/**Supplementary Material**. Further inquiries can be directed to the corresponding author.
